# Biologically plausible reinforcement learning of continuous actions

**DOI:** 10.1186/1471-2202-14-S1-P28

**Published:** 2013-07-08

**Authors:** Jaldert O Rombouts, Pieter R Roelfsema, Sander M Bohte

**Affiliations:** 1 Life Sciences, Centrum Wiskunde en Informatica (CWI), 1098XG, Amsterdam, The Netherlands; 2 Department of Vision & Cognition, Netherlands Institute for Neurosciences, an institute of the Royal Netherlands Academy of Arts and Sciences (KNAW), 1105 BA, Amsterdam, The Netherlands; 3 Department of Integrative Neurophysiology, Centre for Neurogenomics and Cognitive Research (CNCR), VU University, 1081 HV, Amsterdam, The Netherlands; 4 Psychiatry Department, Academic Medical Center (AMC), Amsterdam, The Netherlands

## 

Humans and animals have the ability to perform very precise movements to obtain rewards. For instance, it is no problem at all to pick up a mug of coffee from your desk while you are working. Unfortunately, it is unknown how exactly the non-linear mapping between sensory inputs (e.g. your mug on the retina) and the correct motor actions (e.g. a set of joint angles) are learned by the brain. Here we show how a biologically plausible learning scheme can learn to perform non-linear transformations from sensory inputs to continuous actions based on reinforcement learning.

To arrive at our novel scheme, we built on the idea of attention-gated reinforcement learning (AGREL) [1], a biologically plausible learning scheme that explains how networks of neurons can learn to perform non-linear transformations from sensory inputs to discrete actions (e.g. pressing a button) based on reinforcement learning [2]. We recently showed that the AGREL learning scheme can be generalized to perform multiple simultaneous discrete actions [3], and we now show how this scheme can be further generalized to continuous action spaces. The key idea is that motor areas have feedback connections to earlier processing layers which inform the network about the selected action. Synaptic plasticity is constrained to those synapses that were involved in the decision, and it follows a simple Hebbian rule which is gated by a globally available neuromodulatory signal that codes reward prediction errors. In our novel scheme motor units are situated in a population coding layer that encodes the outcome of the decision process as a bump of activations [4]. This contrasts to our earlier work where single motor units code for actions [1,3]. We show that the synaptic updates perform stochastic gradient descent on the prediction error that results from the combined action-value prediction of all the motor units that encoded the decision. Unlike other reinforcement learning based approaches, e.g. [5], our reinforcement learning rule is powerful enough to learn tasks that require non-linear transformations. The distribution of population centers in the motor layer can also be automatically adapted to task demands, yielding more representational power when actions need to be precise.

We show that the novel scheme can learn to perform non-linear transformations from sensory inputs to motor outputs in a variety of direct reward tasks. The model can explain how visuomotor coordinate transforms might be learned by reinforcement learning instead of semi-supervised learning as used in [6]. It might also explain how humans learn to weigh the accuracy of their movement against the potential rewards and punishments for making inaccurate movements as in the visually guided movement task described in [7].
